# Dental Organizations

**Published:** 1879-09

**Authors:** R. B. Winder


### DENTAL ORGANIZATIONS.

BY PROF. R. B. WINDER.

*Read before the American Dental Convention.*

*Mr. President and Gentlemen of the American Dental Convention:* I have been requested to say something in regard to dental organizations and I have to congratulate myself, not you, upon the opportunity thus afforded me of expressing my views in relation to these matters. I shall, however, be exceedingly brief, as I think the time has come when action, not verbiage, is the order of the day—consequently there is no need for trespassing upon your valuable time in idle talk. The world generally is progressing with gigantic strides; we are living in a progressive age, so much so, that almost everything seems in a state of revolution. Changes are taking place so rapidly with the spirit of the times, that it becomes difficult to reconcile them, and to keep pace with the constantly moving panorama of new born facts and their diverse application. Our profession, which embraces both science and art, must be up and doing. We will not enter into a retrospective view of the means by which dentistry has arrived at its present stand-point of excellence. We are all familiar with the past history of our profession, and need not to have it repeated here. We have to deal with the present and future, not the past.

I think, however, we will all admit without discussion, the premises that society organizations—both local and general—have had much to do with our advancement, and consequently we still need to use these levers to lift us to higher and better things. We thoroughly understand the importance of our state societies, and must therefore labor to keep them up to the highest stand-point. They are the fountains from which the streams of knowledge flow—the laboratories whence the main good comes. The general societies are more like a reservoir intended to receive these blessings as they are poured in from these various rivulets—the grand emporium into which are carried all the valuable accumulations of labor and toil—the granaries where all the ripe fruits of a rich harvest are stored up as common property for future use and reference—a central library open to all—in the language of the illustrious Dr. Atkinson, "a big camp meeting" where all can meet, mingle, and be happy.

It is useless then to discuss the questions of the necessity and usefulness of either local, state, or general dental organizations. We feel quite certain that if these auxiliaries were swept away, dentistry would soon stagnate.

I shall not therefore in this paper say anything about our state organizations, more than that, we must have them, must cherish them, must nourish them; and that each state must in its own wisdom, so arrange and govern its own society, as to bring it up to the necessities of the case.

Regarding general associations, the times demand a national organization, which shall embrace in its membership all who are worthy, and know no North, South, East or West, but whose purpose shall be, "the greatest good to the greatest number."

The advantages which a national organization would give o the whole profession of the United States are as legion, and we may with great propriety, enumerate some of them here.

In the first place, I would say that if the United States are to have, and to hold among the different peoples of the earth, the highly national reputation in dentistry, which has been so earnestly sought and assiduously labored for, it becomes absolutely necessary that we should have a representative and authoritative national organization, endorsed by all the different states through their state societies, as an exponent of our progress, importance, standing and culture. An organization of this sort would certainly carry much more weight and authority, both at home and abroad, than we could possibly expect from our present arrangements. It is the very first principle in military tactics, and I believe the same principle will apply in almost everthing where there is any labor to be performed, any end to be obtained, or any difficulties or obstacles to be overcome and removed—that to win, "forces must be concentrated—not scattered."

By this arrangement we could get all our materials together. All the best intellect and energies of the profession could if necessary, be brought to bear upon one objective point. Such an organization would be your base of supplies—you would have all your resources in hand, and could operate in any direction you might see fit. You would be clothed with national authority and power. We have much talent of one kind or another in the scattered ranks of our profession, it could in this way be collected, brought together and utilized. Such an organization would, of necessity, wipe out all geographical lines and sectional prejudices—each district of country having the same guaranteed rights and privileges—and the end would be the formation of one grand brotherhood, thoroughly united in everything that could tend to promote the present and future welfare and prosperity of dentistry, and in such a harmonious body of representative national men, the highest perfection and attainment in dental art and science would be rapidly realized. This organization would also, from the first, practically relieve us from much inconvenience and annoyance in the fact, that there are many of us members of the three large associations of the country, who would willingly attend all their meetings, and who have for all of them the kindest feelings and best wishes, but who do not like to discriminate in favor of one or the other, and both time and money are wanting to effect a meeting with all. Surely something is due to the comfort and health of the dentist's family, and in his vacation and rest from toil and labor, the constant attendance upon these meetings becomes irksome and unnecessary. If the state societies would do the work which they should do, all we would need would be a national association, where the members of the state societies might occasionally meet. This would not fall hard upon any one, and we could have zealous and earnest meetings, where we could commune, compare old with new, legislate, if necessary, and separate and go back to our homes, and report all the good that had been effected. This organization would serve as a Mecca, from which each weary pilgrim might return fully replenished, and blessed with the wealth of all the newly discovered facts and beauties in art and science, so lavishly bestowed to all true worshipers at such a shrine.

There was a time when wandering tribes in good old patriarchial days, carried with them their government and means of progress. This day has passed. The progress of civilization demands more than this. A country without a government or head is only anarchy. The medical profession has its representative national organization. The scientists have theirs, and why should we hesitate and be alone? We have nothing but praise to bestow upon the present dental organizations; they have fought the good fight and deserve the praise. They have accomplished much good and deserve the respect and confidence of all. But this is no reason why we should not have a national organization. It strikes me that there can be no reasonable objections or opposition to this movement. Some people however, will say: Have we not already a national organization? I would ask them, by way of reply, Could this possibly be so, when we have three so called national organizations—The American Dental Convention, the American Dental Association, and the Southern, (now National Association). Does this look as if we had a national association, you might say we have several. But is there *one* ONE national? We don't have several United States senates, and several houses of congress. We had this condition of things once—two senates and two houses of congress. But was this an out growth of peace and harmony? I did not find it so. There was about that time plenty of hair pulling between the South and her big sister, the North, and the war dogs were unmuzzled, and running around loose, and things were not pleasant, and nobody was happy unless he was a contractor. There must be a head, and the profession requires it, the times demand this national organization. The American Dental Convention, which is the oldest general society in the country, and the

Southern Dental Association saw this two years ago, and passed the necessary resolutions to try and effect it. They made all sorts of overtures to the American Dental Association, and even went so far as to adjourn and meet at Niagara last year, so as to give the American Dental Association no possible or reasonable excuse for not co-operating. The American Dental Association was invited the year before to meet with the American Dental Convention, and the Southern Dental Association, but as I learn from good authority, declined through a misconception of the object in view, so that as the mountain would not to come Mahomet, Mahomet went to the mountain. But the American Dental Association still refused co-operation. We will not say one word against the American Dental Association. It has done much good, and it is the earnest hope and wish of the speaker that it will continue to live and flourish as long as its career of usefulness exists. The formation of a national organization does not militate against the interest of any other associations, and is not meant to destroy but to save.

Now, Mr. President, having argued the necessity of this national organization, we will briefly discuss the principles and general outlines upon which we can form such an organization. The first question that suggests itself to me is, how often and where shall we meet? I would suggest, in reply to this query, that we ought to meet annually—one year in the North, one year in the South, one year in the East, one year in the West and one year in the centre, at Washington, our seat of government. I would meet annually in this way, in order that we may more effectually carry out the object of the organization. "The greatest good to the greatest number." Because by swinging around the circle we adapt our meetings to suit the necessities arising from the scattered condition of our rank and file, and you bring the same high privileges within the easy reach of the most destitute, our gain would be in ultimately having the whole profession with us, and our membership be counted by the thousand Under this arrangement, no one could afford to be left out in the cold. I would also suggest, as a means of retaining this immense membership and gradually utilizing it, that no dues be demanded from a member, except when he was present at the meeting. With some of us, the expense of dues is a very small matter, but there are others—and worthy members of the profession too—who are not so fortunate. This arrangement would at last amount to the same thing and place, the onus of expense upon each geographical section of the country in turn. Every fifth year we should meet at the seat of government. We should obviously do this, in order to present the appearance of a national organization to the rest of the world, and in this year we might occasionally invite an international meeting. Unquestionably for this purpose, our seat of government should be the meeting place, but we have other reasons. It is to be hoped—if this organization should be a success—that it will gradually accumulate a library, scientific apparatus, and perhaps a museum, and we would certainly need some central place to store our archives. It certainly would not look very well to be traveling around with our paraphernalia like a circus or menagerie.

Again it becomes important in its influence upon the government itself, if we ever hope to realize the appointment of dental surgeons to the army and navy of the United States; a necessity which sooner or later must be supplied. For the reasons given, I would then earnestly advise that we should meet as has been suggested, and that these geographical divisions of our territory should be in the commencement marked out and definitely agreed upon and settled, so as to have no room in the future for discussion or contention, and perhaps it would be well to ignore the idea of North, South, East and West, and divide the country into geographical districts; making provision for territory that will, in time, become states. It really seems to me that this would be the most sensible and feasible plan, California is certainly entitled to the same privileges which the other states have, and it looks almost an impossible thing to make a North and East. Politically, these divisions may answer the purpose, but I think a better division can be made to suit us. In regard to the government of this national organization, I would earsently recommend that the officers and executive commi'tee should each year be chosen from the section or district of country where the next meeting takes place, and that the time and place of meeting be also left to the wishes and best judgment of the section in which it meets. Surely, no one would desire to go to New Orleans in the heat of summer, or to Portland, Maine, in the depth of winter. I think, too, that we should always have some one at Washington, to take charge of our archives, etc., and who could act at the same time as corresponding secretary so the whole world, might always know where and to whom to direct any communication for our national organization. As the consummation after this organization shall have gotten into good working order, I would have permanent sections embracing the different subjects pertaining to the science and art of dentistry formed of a limited number of life memberships and selected upon, merit alone for scientific attainment and artistic skill. I would have these positions the posts of honor, and would offer them as a reward to the diligent and meritorious. Such a badge of distinction would be the highest award our profession could boast, and would stimulate young men to labor assiduously and persistently in some one avenue of science or art, hoping in the end to win and wear this honorable distinction. It would become the goal of the highest aspirations. I have thus briefly given you a general outline of the principles upon which this grand organization should be formed, as I think and believe. But the organization itself will depend upon the report of your committees. The detail and minutiæ of government will have to be mapped out by these same committees, and I know and feel that it will take much time and careful consideration to bring every thing into harmonious and proper working order. The question of membership is also to be decided upon. If it can be so arranged, I think it most desirable to have our membership consist of delegates from state societies only. This would compel every state to form a state society or lose its representation, but I would give to all members of state societies the privilege of membership in this national body.

The Southern State Dental Association met in Augusta, Ga., in July, and has carried out in good faith its agreement with this convention to unite with it, in forming a national organization, and has appointed the first Tuesday in September, 1880, to meet with you in the city of New York, for the purpose of consummating this great scheme. The President, Dr. Patrick, of Charleston, South Carolina, has plenary powers to appoint the necessary working committees, etc. I learned on Monday, that the American Dental Association has also appointed a committee, (two of whom are present with us) Prof. Pierce, of Philadelphia, and Dr. McKellops, of St. Louis, Mo., to confer with our committees on this subject. We have a committee to appoint to aid in this great work, and I can only say, where so much is at stake, that I most sincerely hope that the members of our committee will be most carefully selected as men of enlarged views, good judgment, intelligence, energy and free from prejudices. All these qualifications will be necessary and needed for this Herculean task.

I thank you for your very kind attention, and most sincerely hope that our fondest expectations in regard to this great undertaking may be fully realized at our ratification meeting, on the first Tuesday in September, 1880, in the city of New York.

